# Comparative Analysis of Dalbavancin versus Other Antimicrobial Options for Gram-Positive Cocci Infections: Effectiveness, Hospital Stay and Mortality

**DOI:** 10.3390/antibiotics10111296

**Published:** 2021-10-24

**Authors:** Mar de Pablo-Miró, Sergi Pujol-Ruiz, Simona Iftimie, María del Mar Arenas-Miras, Inmaculada López-Montesinos, Xavier Duran-Jordà, Albert Anglès, Santiago Grau, Juan P. Horcajada

**Affiliations:** 1 Department of Infectious Diseases, Hospital del Mar, Institut Hospital del Mar d’Investigacions Mèdiques (IMIM), 08003 Barcelona, Spain; mardepablomiro@gmail.com (M.d.P.-M.); sergi.pujol316@gmail.com (S.P.-R.); marenas@psmar.cat (M.d.M.A.-M.); ilopezmontesinos@psmar.cat (I.L.-M.); 2 Faculty of Health and Life Sciencies, Universitat Pompeu Fabra, 08003 Barcelona, Spain; sgrau@psmar.cat; 3 Infectious Diseases Service, Hospital Universitari Sant Joan de Reus, 43204 Reus, Spain; smiftimie@grupsagessa.com; 4 Statistics Service, Institut Hospital del Mar d’Investigacions Mèdiques (IMIM), 08003 Barcelona, Spain; xduran@imim.es; 5 Documentation Service, Hospital del Mar, 08003 Barcelona, Spain; aangles@parcdesalutmar.cat; 6 Pharmacy Service, Hospital del Mar, 08003 Barcelona, Spain; 7 REIPI (Red Española de Investigación en Patologías Infecciosas)—ISCII (Instituto de Salud Carlos III) Spanish Network for Research in Infectious Diseases, 28029 Madrid, Spain

**Keywords:** dalbavancin, hospital stay, effectiveness, mortality, gram-positive, multidrug-resistant gram-positive cocci, methicillin-resistant *Staphylococci*

## Abstract

Dalbavancin is a new antibiotic that is effective against Gram-positive microorganisms, including methicillin-resistant *Staphylococci*, and offers the possibility of administering intravenous therapy once weekly in an ambulatory setting. We conducted a multicenter observational case-control study, comparing all patients who received dalbavancin (cases) with hospitalized patients who were treated instead with daptomycin, linezolid or vancomycin (controls), based on clinical diagnosis, main microorganism involved, and age. The primary outcome was the length of hospital stay after starting the study antimicrobial. Secondary outcomes were 7-day and 30-day efficacy, 30-day mortality, 90-day recurrence, 90-day and 6-month hospitalization, presence of adverse events and healthcare-associated infections; 161 patients (44 cases and 117 controls) were included. Bivariate analysis showed that dalbavancin reduced the total length of hospital stay (*p* < 0.001), with fewer 90-day recurrences (*p* = 0.005), 6-month hospitalizations related to the same infection (*p* = 0.004) and non-related hospitalizations (*p* = 0.035). Multivariate analyses showed that length of hospital stay was significantly shorter in patients treated with dalbavancin (−12.05 days 95% CI [−17.00, −7.11], *p* < 0.001), and 30-day efficacy was higher in the dalbavancin group (OR 2.62 95% CI [1.07, 6.37], *p* = 0.034). Although sample size of the study may be a limitation, we can conclude that Dalbavancin is a useful antimicrobial drug against Gram-positive infections, including multidrug-resistant pathogens, and allows for a remarkable reduction in length of hospital stay with greater 30-day efficacy.

## 1. Introduction

Gram-positive infections remain an important cause of infection, exhibiting high burden in terms of morbidity and mortality. Multidrug-resistant Gram-positive pathogens are still a major public health concern, both in community-acquired and healthcare-associated infections [1,2,3,4]. Due to the high proportion of Gram-positive infections caused by β-lactam-resistant microorganisms, vancomycin, daptomycin and linezolid are among the most frequently used therapeutic options [5]. Since these antimicrobial therapies have some limitations (those arising from daily intravenous administration), there remains a demand for optimal antibiotic therapies that not only guarantee a good clinical and microbiological profile, but also good therapy compliance, a good safety profile, quality of life and cost-effectiveness.

Dalbavancin is a semisynthetic second-generation lipoglycopeptide antimicrobial that is effective against Gram-positive microorganisms, including multi-drug resistant rods [6]. Interestingly, it has a half-life of 7.5–9 days, which offers the possibility of intravenous administration once a week in ambulatory therapy [6]. So far, dalbavancin has been approved in the USA and Europe to treat moderate-to-severe acute bacterial skin and skin-structure infections (ABSSSI), and has shown accelerated discharge of hospitalized patients, non-inferior efficacy and fewer adverse events [7,8]. This drug regimen is unique, and lack of experience prompts the need to conduct more studies, especially in real-life scenarios involving other clinical diagnoses.

Our hypothesis is that once-weekly intravenous administration of dalbavancin is a valid alternative to other antibiotics available for the treatment of Gram-positive cocci infections, including multidrug-resistant microorganisms, which prevents hospitalization and accelerates hospital discharge.

The main purpose of this study is to analyze the clinical impact of dalbavancin treatment on patients, focusing on clinical outcome, mortality, length of hospital stay, and the presence of adverse events and healthcare-associated infections and to compare it with other antimicrobial therapies used for the treatment of similar clinical diagnosis infections.

## 2. Results

### 2.1. Patient Demographics and Disease Characteristics

A total of 161 patients were included: 44 cases (26 from Hospital del Mar and 18 from Hospital Universitari Sant Joan) and 117 controls (74 Hospital del Mar and 43 Hospital Universitari Sant Joan).

Demographic and baseline characteristics between groups were largely similar (Table 1). Patients treated with dalbavancin presented a higher Charlson Comorbidity Index (CCI) score than those treated with the other antimicrobial therapies, this difference being statistically significant (4.0 [IQR 2.0–6.0] vs. 3.0 [IQR 1.0–5.0], *p* = 0.043). Intravenous drug users were more frequently treated with dalbavancin, although differences were not statistically significant (9.1% vs. 3.4%, *p* = 0.216).

Disease characteristics were similar between groups. Overall, the most frequent type of infection in patients treated with dalbavancin was vascular infection and/or endocarditis (27.3%). No statistically significant differences were observed in the distribution of clinical diagnosis compared to the control group (*p* = 0.998). Among patients treated with dalbavancin, the most frequent microorganism was *Staphylococcus aureus* (50%), which was oxacillin-susceptible in 35 controls and 13 cases, and oxacillin-resistant in 38 controls and 9 cases, with no statistically significant differences between groups (*p* = 0.316).

Among patients treated with dalbavancin, 15.9% received the antibiotic empirically and 84.1% as pathogen-targeted therapy versus 41.9% and 54.7% respectively in the control group. These differences were statistically significant (*p* < 0.001).

### 2.2. Clinical Outcome

Clinical outcome variables are shown in Table 2. In the dalbavancin group, a significantly shorter length of hospital stay was observed during the period of the studied antimicrobial treatments (median days 0.0 [0.0–0.0] vs. 13.0 [6.0–27.0], *p* ≤ 0.001). Total length of hospital stay was also lower in the dalbavancin group (median days 10.5 [0.0–16.0] vs. 18.0 [9.0–36.0], *p* < 0.001]. Seven-day efficacy rate (81.8% vs. 88%, *p* = 0.312) was slightly lower, and 30-day efficacy rate (81.8% vs. 66.7%, *p* = 0.079) was higher in the dalbavancin group. Ninety-day recurrence rate (2.3% vs. 18.8%, *p* = 0.005) and related 6-month hospitalization rate (0% vs. 15.4%, *p* = 0.004) were lower in the dalbavancin group. Non-related 6-month hospitalization rate (27.3% vs. 12.8%, *p* = 0.035) was higher in the dalbavancin group. Dalbavancin presented fewer adverse events and healthcare-associated infections, but differences were not statistically significant.

Bivariate analyses of factors related to length of hospital stay are shown in Table 3. In the multivariate regression model of the length of hospital stay, only the antibiotic used (dalbavancin versus others) proved to be an independent factor.

Bivariate analyses of factors related to 7-day efficacy showed that only kidney insufficiency was statistically significantly lower in the failure group (74.1 [50.5, 92.0] vs. 41.0 [25.7, 74.5], *p* = 0.027) and SAPS II score was higher in the failure group (35.0 [26.0–39.0] vs. 28 [23.0 vs. 37.0], *p* = 0.071), but was not statistically significant. There were no associated factors in the multivariate regression model of 7-day efficacy. These data are not shown in the tables.

Bivariate analyses of factors related to 30-day efficacy showed no statistically significant differences. The adjusted model of multivariate regression showed that dalbavancin had increased 30-day efficacy (OR 2.62 95% CI [1.07, 6.37], *p* = 0.034). Low Charlson Comorbidity Index was also identified as an independent protective factor (OR 0.83 95% CI [0.72, 0.96], *p* = 0.013). These data are not shown in the tables.

Bivariate and multivariate analyses of factors related to 30-day crude mortality are shown in Table 4. No factors were associated in the multivariate regression model of 30-day global mortality. Dalbavancin presented a lower 30-day global mortality rate, but this was not statistically significant (OR 0.14, 95% CI 0.02 to 1.3; *p* = 0.078).

## 3. Discussion

Overall, this study shows that once weekly intravenous administration of dalbavancin as an ambulatory therapy is an effective alternative for the treatment of Gram-positive cocci infections, including those that are methicillin-resistant *Staphylococci*. Multiple reports have drawn attention to the worldwide increase in Gram-positive cocci infections in recent years [4], as well as to the limitations of conventional antimicrobials (such as those arising from daily intravenous administration). There has been growing interest in particular in novel antibiotics such as dalbavancin, which is proving to be a valid alternative that not only guarantees a good clinical and microbiological profile [9], but also good compliance [10], a good safety profile [9,11], quality of life [12], and cost effectiveness [8,12,13]. The present study found that a dalbavancin-based treatment reduced the length of hospital stay, 90-day recurrence and related 6-month hospitalization, and had a higher 30-day efficacy. Dalbavancin safety was similar to conventional treatments, thus highlighting dalbavancin as a safe and valid alternative for the treatment of Gram-positive cocci infections.

The present study found that the characteristics of patients treated with dalbavancin were heterogenous and consistent with those found in previous studies that included off-label uses of dalbavancin [14] in terms of age, gender, comorbidities, clinical diagnosis and types of microorganism. Poliseno et al. found that patients treated with dalbavancin at their center had a mean age of 61, 68% were male, the median Charlson Comorbidity Index score was 3, and 72% of *Staphylococci* in their sample (34% oxacillin-susceptible and 38% resistant) [14]. Morrisette et al. and Wilke et al. found similar patient characteristics [15,16]. These results were similar to those obtained in our study. Interestingly, in our study, the main type of infection treated with dalbavancin was vascular infection and/or endocarditis, not skin and acute bacterial skin and skin structure infections (ABSSSI), which is the only indication approved by the European Medicines Agency [7]. A higher percentage of IVDUs was also found in the dalbavancin group (9.1% vs. 3.4%), although this was not statistically significant (*p* = 0.216). Morrisette et al. [15] also found greater eligibility for dalbavancin in IVDU patients, which represents a useful solution for reducing the infection risks of long-term central lines and abuse concerns, whilst increasing treatment compliance.

The baseline characteristics of the two groups in our study were also similar, with optimal case-control matching, although the mean Charlson Comorbidity Index of those patients treated with dalbavancin was higher than that of hospitalized patients who received conventional antimicrobial therapies, indicating that dalbavancin can be successfully used in complex patients [13]. The mean SAPS II of patients treated with dalbavancin was also slightly higher but not statistically significant, showing that dalbavancin is an option for treating more severe infections [17].

Our study found that dalbavancin was successfully prescribed for the treatment of many different types of microorganisms, including methicillin-resistant *Staphylococci*, and on- and off-label uses. Both factors were consistent with other studies involving bloodstream infection and infectious endocarditis [18], osteomyelitis [19,20], and joint infections [21], among many others [9,13,22].

In our experience, length of hospital stay with dalbavancin was considerably shorter, which coincides with other studies [9,14,20]. Therapeutic approaches allowing for early patient discharge have been of particular interest during the SARS-CoV-2 pandemic, when shortages of available hospital beds and the risks of infection while hospitalized have been critical factors. Dalbavancin also showed higher 30-day efficacy than comparators. The clinical success rate of 81.8% at days 7 and 30 is similar to results obtained in other studies including on-label [23] and off-label prescriptions [9,14]. In our study, a protective effect for 30-day global mortality was observed in the dalbavancin group (OR = 0.138), which is close to the threshold of statistical significance (*p* = 0.078), probably due to the low incidence of this event, and is consistent with other studies [24]. Both 90-day recurrence and 6-month hospitalizations (related and non-related) were also shown to be lower, as has been found in other studies [25]. Our study also assessed safety and tolerability and a tendency towards fewer adverse events in the dalbavancin group was observed, similar to other studies [8,23]. All were of low-to-moderate severity, as in previous series [18,23]. Finally, our study also showed a reduction in healthcare-associated infections, which could be explained by the shorter period of hospital stay.

Veve et al. recently conducted a similar study, also in a real-life scenario, comparing dalbavancin with standard-of-care or vancomycin and daptomycin in a total of 215 patients with osteoarticular infection, infective endocarditis, or another bloodstream infection [25]. They studied several endpoints including 90-day infection-related readmission, time to infection-related readmission, adverse events and all-cause readmission and mortality. As in our study, they also showed a reduction in length of hospital stay in readmission rates with dalbavancin. However, we also assessed antimicrobial efficacy, showing that it was higher at 30-days with dalbavancin.

Our study has the following limitations. First, the relatively small sample size. Second, the retrospective design, although real-life, involved a non-randomized process assigned according to the clinician’s judgement. Treatment in both the case and the control group was not homogenous in terms of duration and dosing, and a larger proportion of patients in the control group received empirical treatment. In addition, the outcome variables were obtained retrospectively from clinical records and required interpretation by the researchers. Third, the attempt to cover all types of clinical diagnosis and types of micro-organism resulted in a heterogenous sample that was analyzed as a whole, without establishing sub-groups.

Notwithstanding the limitations mentioned above, we believe our study has several strengths and originality with respect to the published literature. The inclusion of two centers, multiple clinical diagnoses and multiple microorganisms enhances representativeness and reflects the real-life situation of many acute care hospitals and the wide range of possible applications of dalbavancin. Despite this heterogeneity, our restrictive matching criteria allowed for very similar characteristics between groups. Finally, the analysis of multiple outcome variables made our study more robust.

Further studies in larger groups of patients should be performed, involving on- and off-label uses of dalbavancin, and should be randomized if possible. However, available data from real life studies like ours confirms dalbavancin to be a safe and efficacious option against Gram-positive infections, including multidrug resistant, reducing hospital stay and readmission rates.

## 4. Materials and Methods

A multicenter, observational case-control study was conducted at the Hospital del Mar (a 420-bed tertiary care university hospital in Barcelona, Spain) and the Hospital Universitari Sant Joan de Reus (a 352-bed tertiary care university hospital in Reus, Spain). The case group (*n* = 44) included all adult patients who received at least one dose of dalbavancin, prescribed by their attending physician, between November 2015 and December 2019. The control group included adult patients who could have been treated with dalbavancin but received another antimicrobial therapy (linezolid, daptomycin or vancomycin) during the same time period, at the discretion of the attending physician (*n* = 117). The case-control ratio was 1:3. The matching criteria were clinical diagnosis, main pathogen involved, and age. When the three criteria were applied together, the initial groups were not large enough and the criterion for control inclusion was relaxed to the same clinical diagnosis, a similar main pathogen, at the investigator’s discretion, and a wider age range. Nevertheless, the restrictive matching criteria did not always permit 3 controls per case. To minimize cases without controls, 4 cases from the Hospital Universitari Sant Joan de Reus for which no controls could be found in the same hospital were paired with 4 controls from the Hospital del Mar.

Clinical data were retrospectively collected from electronic medical charts. Baseline characteristics included demographics, comorbidities, Charlson Comorbidity Index [26], risk of multidrug-resistant infection, clinical diagnosis of the main infection, microorganism involved, presence of concomitant infection, assessment of clinical severity, Simplified Acute Physiology Score (SAPS) II [27], duration of antibiotic treatment, route of administration and type of antibiotic treatment (prophylactic, anticipated, empiric or directed) and need for management of site (surgery, debridement and/or drainage). Assessments of clinical outcomes were retrospectively collected from medical charts and included length of hospital stay (global and with the antimicrobial therapy studied), 7-day and 30-day efficacy, 90-day recurrence, 90-day hospitalization, 6-month hospitalization (related and non-related), 30-day mortality (global, related, and non-related), presence of adverse events and presence of healthcare-associated infections [28]. The primary outcome was the length of hospital stay with the antimicrobial therapy of the study; 7- and 30-day efficacy were based on clinical records and measured as categorical items, at the discretion of the researcher. The categories were: (1) Complete healing: negative samples were obtained and/or clinical records showed resolution of infection; (2) Improvement: information about healing or failure on clinical records was unclear, but favorable evolution was as expected; or (3) Failure: positive samples were obtained and/or clinical records showed explicit failure and/or clinical records showed a worsening of the expected evolution of the infection; 7 and 30-day efficacy were finally treated as dichotomous variables and were categorized according to the natural evolution of the infection under antimicrobial treatment: 7-day efficacy was categorized as failure vs. non-failure (included complete healing and improvement) and 30-day efficacy as cured vs. non-cured (included improvement and failure).

Dalbavancin was administered as a single or multiple intravenous dose of 1000 mg or 1500 mg over 30 min. Dosing as well as length of treatment were chosen by the pre-scribing physician. Dose adjustment was required only for patients with severe renal dysfunction (creatinine clearance (CLCr) < 30 mL/min). Dosing and length of therapy of the other antimicrobial therapies were chosen by the prescribing physician according to protocols.

Routine identification and susceptibility testing of causative microorganisms were per-formed using automated systems (Vitek-2^®^ (BioMérieux, Marcy-l’Étoile, France) for blood cultures, and the MicroScan^®^ WalkAway (Beckman-Coulter, Brea, CA, USA) for other types of sample) and interpreted in accordance with the standards defined by the European Committee on Antimicrobial Susceptibility Testing (EUCAST).

Sample size was determined through a power calculation accepting an alpha risk of 0.05 and a beta risk of 0.2 in a two-sided test, using length of hospital stay as the main outcome. Taking a ratio of 1:3 (dalbavancin versus other antimicrobial therapies), 33 subjects were required in the dalbavancin group and 99 in the other antimicrobial therapy group (daptomycin, linezolid, and vancomycin) to detect a statistically significant difference in hospital stay of 4 days or more. The common standard deviation is assumed to be seven days. A drop-out rate of 0% was anticipated.

Categorical variables were presented as numbers of cases and percentages, and continuous variables as a median and interquartile range (IQR). Comparisons between groups were tested by the *t*-test or Mann–Whitney U test; the Pearson’s chi-squared or Fisher’s exact test were used to compare categorical variables, as appropriate. Correlations between continuous variables were evaluated using Spearman’s rank correlation coefficients.

A multivariate logistic regression model using a backward stepwise selection examined the independent variables associated with seven and 30-day efficacy and 30-day mortality. Results were expressed as OR and a confidence interval. Length of stay was evaluated by multivariate median regression to deal with the lack of normality of dependent variables. Results were expressed as a median and confidence interval. The interpretation of these coefficients was analogous to the interpretation of coefficients in multiple linear regression. All *p*-values were 2-tailed and statistical significance was <0.05. Statistical analysis was performed using STATA 15.1.

## 5. Conclusions

Although sample size may be a limitation, this study shows that administering dalbavancin intravenously once a week is an effective alternative for the treatment of Gram-positive cocci infections, including multidrug resistance and severe infections, and complex patients. A dalbavancin-based treatment reduces the length of hospital stay, 90-day recurrence and 6-month-related hospitalizations and has higher 30-day efficacy and similar safety when compared with conventional treatments.

This study was presented at the XXIII Congreso de la Sociedad Española de Enfermedades Infecciosas y Microbiología Clínica, held on-line in July 2021.

## Figures and Tables

**Table 1:** Baseline characteristics for patients treated with dalbavancin (cases) and those treated with other conventional antimicrobial therapies (daptomycin, linezolid, and vancomycin) (controls).

Clinical Variable	Daptomycin, Linezolid or Vancomycin (*n* = 117)	Dalbavancin (*n* = 44)	*p*-Value
Hospital in charge			0.628
Hospital del Mar	74 (63.2)	26 (59.1)	
Hospital Universitari Sant Joan de Reus	43 (36.8)	18 (40.9)	
Age, y, m (IQR)	70.0 (57.0, 77.0)	71 (53.0, 80.5)	0.644
Male sex	71 (60.7)	23 (52.3)	0.372
Charlson Index, m (IQR)	3.0 (1.0, 5.0)	4.0 (2.0, 6.0)	0.043
Cardiac disease	42 (35.9)	15 (34.1)	0.856
Chronic kidney disease	26 (22.2)	6 (13.6)	0.272
Respiratory disease	22 (18.8)	12 (27.3)	0.280
Diabetes mellitus	39 (33.3)	13 (29.5)	0.708
Neurological disease	12 (10.3)	1 (2.3)	0.116
Gastrointestinal disease	13 (11.1)	4 (9.1)	1.000
Liver disease	4 (3.4)	0 (0.0)	0.576
HIV	3 (2.6)	2 (4.5)	0.615
Active solid neoplasia	14 (12.0)	4 (9.1)	0.781
Active hematologic neoplasia	5 (4.3)	3 (6.8)	0.685
Intravenous drug user	4 (3.4)	4 (9.1)	0.216
Risk of multidrug-resistant infection			
Surgery in previous 3 months	22 (18.8)	13 (29.5)	0.197
Hospitalization or medical appointment in previous 3 months	75 (64.1)	23 (52.3)	0.205
Antibiotic administration in previous 3 months	45 (38.5)	23 (52.3)	0.114
Source of main infection			0.998
Vascular and/or endocarditis	32 (27.4)	12 (27.3)	
Skin and soft tissue	25 (21.4)	9 (20.5)	
Osteoarticular	19 (16.2)	7 (15.9)	
Prosthesis	18 (15.4)	6 (13.6)	
Bacteriemia	8 (6.8)	3 (6.8)	
Other (UTI, prostatitis or abdominal infection)	15 (12.8)	7 (15.9)	
Main microorganism			0.316
Not isolated	13 (11.1)	5 (11.4)	
Oxacillin-resistant *Staphylococcus* spp.	38 (32.5)	9 (20.5)	
Oxacillin-susceptible *Staphylococcus* spp.	35 (29.9)	13 (29.5)	
*Streptococcus* spp.	6 (5.1)	7 (15.9)	
*Enterococcus* spp.	17 (14.5)	6 (13.6)	
Other	8 (6.9)	4 (9.1)	
Presence of concomitant infection	33 (28.2)	7 (15.9)	0.151
SAPS II, m (IQR)	28.0 (23.0, 37.0)	34.5 (23.0, 37.5)	0.311
Serum Creatinine concentration, m(IQR)	0.9 (0.7, 1.3)	0.8 (0.7, 1.3)	0.283
CKD-EPI, m (IQR)	69.5 (39.0, 90.0)	74.6 (50.9, 99.9)	0.116
Treatment with study antibiotic			
Days of treatment, m (IQR)	7.0 (5.0, 14.0)	14.0 (14.0, 30.0)	<0.001
Type of treatment			<0.001
Empirical	53 (45.3)	7 (15.9)	
Targeted	64 (54.7)	37 (84.1)	
Management of infectious site (surgery, debridement and/or drainage)	53 (45.3)	15 (34.1)	0.215

Data are presented as *n* (%), unless otherwise specified. Abbreviations: m (median), IQR (interquartile range), SAPS-II (Simplified Acute Physiology Score), CKD-EPI (Chronic Kidney Disease Epidemiology Collaboration), d (days), y (years). *p*-Values < 0.05 are written in bold.

**Table 2:** Bivariate analysis of outcome variables for patients treated with dalbavancin (cases) versus those treated with other conventional antimicrobial therapies (daptomycin, linezolid, and vancomycin) (controls).

Outcome Variable	Daptomycin, Linezolid or Vancomycin (*n* = 117)	Dalbavancin (*n* = 44)	*p*-Value
Total length of hospital stay, m (IQR), d	18.0 (9.0, 36.0)	10.5 (0.0, 16.0)	<0.001
Length of hospital stay, m (IQR) since onset of treatment with study antibiotic, d	13.0 (6.0, 27.0)	0 (0.0, 0.0)	<0.001
7-day efficacy (non-failure)	103 (88.0)	36 (81.8)	0.312
30-day efficacy (cured)	78 (66.7)	36 (81.8)	0.079
90-day recurrence	22 (18.8)	1 (2.3)	0.005
90-day hospitalization	29 (24.8)	7 (15.9)	0.291
Related 6-month hospitalization	18 (15.4)	0 (0.0)	0.004
Non-related 6-month hospitalization	15 (12.8)	12 (27.3)	0.035
30-day global mortality	12 (10.3)	1 (2.3)	0.116
Related 30-day mortality	6 (5.1)	0 (0.0)	0.190
Non-related 30-day mortality	6 (5.1)	1 (2.3)	0.675
Adverse events *	5 (4.3)	1 (2.3)	1.000
Healthcare-associated infections	9 (7.7)	2 (4.5)	0.729

Data are presented as *n* (%), unless otherwise specified. Abbreviations: m (median), IQR (interquartile range), d (days). * Adverse events detected in patients treated with daptomycin, linezolid or vancomycin were medullary toxicity (3), diarrhea caused by *Clostridium difficile* (1) and vomiting (1); in patients treated with dalbavancin, medullary toxicity (1). *p*-Values < 0.05 are written in bold.

**Table 3:** Bivariate and multivariate analyses of factors related to the length of hospital stay.

Clinical Variable	Bivariate Analyses		Multivariate Analyses	
	Median Length of Hospital Stay (IQR) (Days), *p*-Value		Median Difference in Hospital Stay (95% CI) (Days) *p*-Value	
Antibiotic		<0.001		<0.001
Daptomycin, linezolid or vancomycin	13.0 (6.0, 27.0)		−12.1 (−17.0, −7.1)	
Dalbavancin	0.0 (0.0, 0.0)			
Age, y, ρ	ρ 0.012	0.881		
Sex		0.457		
Male	7.0 (0.0, 19.0)			
Female	9.0 (0.0, 19.0)			
Charlson Index, ρ	ρ 0.020	0.806	−0.07 (−0.3, 0.15)	0.135
Cardiac disease		0.327		
No	7.0 (0.0, 17.5)			
Yes	9.0 (1.0, 20.0)			
Chronic kidney disease		0.091		
No	7.0 (0.0, 16.0)			
Yes	14.0 (2.5, 31.5)			
Respiratory disease		0.970		
No	7.0 (0.0, 19.0)			
Yes	9.5 (0.0, 20.0)			
Diabetes mellitus		0.016	4.38 (−0.75, 9.5)	0.094
No	7.0 (0.0, 14.0)			
Yes	13.5 (2.5, 31.0)			
Neurological disease		0.240		
No	7.5 (0.0, 20.0)			
Yes	11.0 (6.0, 15.0)			
Gastrointestinal disease				
No	7.5 (0.0, 18.0)			
Yes	10.0 (0.0, 44.0)			
Liver disease		0.009		
No	8.0 (0.0, 19.0)			
Yes	33.0 (27.0, 68.5)			
HIV		0.302		
No	8.0 (0.0, 20.0)			
Yes	0.0 (0.0, 14.0)			
Active solid neoplasia		0.437		
No	8.0 (0.0, 21.0)			
Yes	5.0 (0.0, 16.0)			
Active hematologic neoplasia		0.102		
No	8.0 (0.0, 20.0)			
Yes	1.0 (0.0, 9.5)			
Intravenous drug user		0.068		
No	9.0 (0.0, 20.0)			
Yes	2.0 (0.0, 6.0)			
Risk of multidrug-resistant infection				
Surgery in previous 3 months		0.711		
No	8.0 (0.0, 19.0)			
Yes	9.0 (0.0, 28.0)			
Hospitalization or medical appointment in previous 3 months		0.946		
No	8.0 (0.0, 20.0)			
Yes	8.0 (0.0, 19.0)			
Antibiotics in previous 3 months		0.938		
No	8.0 (1.0, 16.0)			
Yes	8.5 (0.0, 21.0)			
Source of main infection		0.008		
Vascular and/or endocarditis	9.0 (0.0, 26.5)			
Skin and soft tissue	5.5 (0.0, 20.0)			
Osteoarticular	11.5 (7.0, 27.0)			
Prosthesis	13.5 (4.5, 30.5)			
Bacteremia	9.0 (0.0, 14.0)			
Other (UTI, prostatitis or abdominal infection)	1.5 (0.0, 5.0)			
Main microorganism		0.047		
Not isolated	7.5 (0.0, 13.0)			
Oxacillin-resistant *Staphylococcus* spp.	14.0 (4.0, 36.0)			
Oxacillin-susceptible *Staphylococcus* spp.	9.0 (0.0, 21.0)			
*Streptococcus* spp.	6.0 (0.0, 27.0)			
*Enterococcus* spp.	5.0 (0.0, 14.0)			
Other Gram-positive	0.0 (0.0, 3.0)			
Gram-negative bacilli	14.0 (14.0, 14.0)			
Presence of concomitant infection		0.168		
No	7.0 (0.0, 17.0)			
Yes	11.5 (2.5, 28.0)			
SAPS II, ρ	ρ −0.068	0.410	−0.07 (−0.3, 0.15)	0.527
Creatinine concentration, ρ	ρ 0.059	0.484		
CKD-EPI, ρ	ρ −0.115	0.265		
Treatment with study antibiotic				
Days of treatment, ρ	ρ 0.063	0.446		
Route of administration		0.212		
Intravenous	7.0 (0.0, 19.5)			
Oral	10.0 (4.0, 16.0)			
Enteral	203.0 (203.0, 203.0)			
Mixed regimen	7.0 (3.0, 60.0)			
Management of source (surgery, debridement and/or drainage)		<0.001	4.38 (−0.3, 9.04)	0.066
No	5.0 (0.0, 14.0)			
Yes	13.0 (4.5, 31.5)			

In the bivariate analyses, data are presented as median (interquartile range), unless otherwise specified. Quantitative continuous variables were related with median hospital stay by means of Spearman correlation coefficient (ρ). In the multivariate analyses, differences are expressed as differences in median values (95% CI). Abbreviations: m (median), IQR (interquartile range), SAPS-II (Simplified Acute Physiology Score), CKD-EPI (Chronic Kidney Disease Epidemiology Collaboration), d (days), y (years). *p*-Values < 0.05 are written in bold.

**Table 4:** Bivariate and multivariate analyses of factors related to 30-day mortality.

	Bivariate Analyses			Multivariate Analyses	
Clinical Variable	30-Day Survival (*n* = 148)	30-day Mortality (*n* = 13)	*p*-Value	OR (95% CI)	*p*-Value
Antibiotic			0.116		
Dalbavancin	43 (97.73)	1 (2.27)		0.14 (0.02, 1.25)	0.078
Daptomycin, linezolid or vancomycin	105 (89.74)	12 (10.26)			
Age, m (IQR), y	70.0 (56.0, 78.0)	75.0 (59.0, 77.0)	0.384		
Male sex	87 (92.55)	7 (7.44)	0.775		
Charlson Index, m (IQR)	3.0 (1.0, 5.0)	3.0 (2.0, 5.0)	0.418	1.10 (0.86, 1.41)	0.436
Cardiac disease	51 (89.47)	6 (10.53)	0.546		
Chronic kidney disease	26 (81.25)	6 (18.75)	0.024		
Respiratory disease	32 (94.12)	2 (5.88)	0.738		
Diabetes mellitus	48 (92.31)	4 (7.69)	1.000		
Neurological disease	13 (100.00)	0 (0.00)	0.602		
Gastrointestinal disease	14 (82.35)	3 (17.65)	0.143		
Liver disease	3 (75.00)	1 (25.00)	0.288		
HIV	4 (80.00)	1 (20.00)	0.347		
Active solid neoplasia	13 (8.8)	5 (38.5)	0.007	3.51 (0.97, 14.12)	0.077
Active hematologic neoplasia	7 (87.50)	1 (12.50)	0.498		
Intravenous drug user	7 (87.50)	1 (12.50)	0.498		
Risk of multidrug- resistant infection					
Surgery in previous 3 months	34 (97.14)	1 (2.86)	0.301		
Hospitalization or medical appointment in previous 3 months	88 (89.80)	10 (10.20)	0.252		
Antibiotic in previous 3 months	64 (94.11)	4 (5.88)	0.560		
Source of main infection			0.241		
Vascular and/or endocarditis	38 (86.36)	6 (13.63)			
Skin and soft tissue	32 (94.12)	2 (5.88)			
Osteoarticular	25 (96.15)	1 (3.85)			
Prosthesis	24 (100.00)	0 (0.00)			
Bacteriemia	9 (81.81)	2 (18.18)			
Other (UTI, prostatitis or abdominal infection)	20 (90.91)	2 (9.09)			
Main microorganism			0.664		
Not isolated	18 (100.00)	0 (0.00)			
Oxacillin-resistant *Staphylococcus* spp.	43 (91.49)	4 (8.51)			
Oxacillin-susceptible *Staphylococcus* spp.	42 (87.50)	6 (12.50)			
*Streptococcus* spp.	13 (100.00)	0 (0.00)			
*Enterococcus* spp.	21 (91.30)	2 (8.70)			
Other Gram-positive	10 (90.91)	1 (9.09)			
Gram-negative bacilli	1 (100.00)	0 (0.00)			
Presence of concomitant infection	37 (92.50)	3 (7.50)	1.000		
SAPS II, m (IQR)	30.0 (22.5, 36.5)	36.0 (30.0, 48.0)	0.005	1.05 (0.99, 1.12)	0.105
Creatinine concentration, m (IQR)	0.9 (0.7, 1.3)	1.3 (0.7, 1.6)	0.348		
CKD-EPI, m (IQR)	72.0 (43.5, 90.5)	51.5 (35.0, 67.0)	0.192		
Treatment with study antibiotic					
Days of treatment, m (IQR)	12.0 (5.0, 20.5)	7.0 (5.0, 10.0)	0.112		
Management of infection source (surgery, debridement and/or drainage)	66 (97.06)	2 (2.94)	0.045	0.38 (0.07, 2.01)	0.253

In the bivariate analyses, data are presented as *n* (%), unless otherwise specified. In the multivariate analyses, data are expressed as odds ratios (95% CI). Abbreviations: m (median), IQR (interquartile range), OR (odds ratio), SAPS-II (Simplified Acute Physiology Score), CKD-EPI (Chronic Kidney Disease Epidemiology Collaboration), d (days), y (years). *p*-Values < 0.05 are written in bold.

## Data Availability

The data presented in this study are available on request from the corresponding author (J.P.H.) upon reasonable request.
